# PREventing Delayed Graft Function by Driving Immunosuppressive InduCtion Treatment (PREDICT-DGF): study protocol for a randomized controlled trial

**DOI:** 10.1186/s13063-015-0807-x

**Published:** 2015-06-23

**Authors:** Marion Chapal, Yohann Foucher, Monique Marguerite, Karine Neau, Emmanuelle Papuchon, Pascal Daguin, Emmanuel Morélon, Georges Mourad, Elisabeth Cassuto, Marc Ladrière, Christophe Legendre, Magali Giral

**Affiliations:** ITUN and Inserm U1064, Nantes University, 30 Boulevard Jean Monnet, Nantes, 44035 France; SPHERE (EA4275), Nantes University, Nantes, France; Délégation à la recherche clinique et à l’innovation, CHU Nantes, Nantes, France; Néphrologie, Transplantation et Immunologie Clinique, Hôpital Edouard Herriot, Lyon, France; Service de Néphrologie, Dialyse et Transplantation, Hôpital Lapeyronie and Université Montpellier I, Montpellier, France; Service de Néphrologie, Hôpital Pasteur, Nice, France; Service de Transplantation Rénale, CHU Brabois, Nancy, France; Service de Transplantation Rénale et de Soins Intensifs, Hôpital Necker, APHP and Universités Paris Descartes et Sorbonne Paris Cité, Paris, France; Centre d’Investigation Clinique en Biothérapie, Nantes University Hospital, Nantes, France; CENTAURE Fondation, Nantes, France

**Keywords:** Delayed graft function, Kidney transplantation, Randomized clinical trial, Anti-thymocyte globulins

## Abstract

**Background:**

In kidney transplantation, the use of Anti-Thymocyte Globulins (ATG) as induction therapy has been described as a possible treatment for reducing the prevalence of Delayed Graft Function (DGF). ATG possesses pharmaceutical proprieties that could help control the lesions caused by ischemia reperfusion injury. However, other studies have questioned this potential protective effect. We hypothesized that the benefits related to ATG for reducing DGF prevalence may be higher and more consistently recognized if only patients with high DGF risk are considered. We recently proposed a scoring system entitled DGFS (Delayed Graft Function Score) for such stratification of kidney transplant recipients according to their risk of DGF. Using the DGFS calculation, we aim to determine whether a short course of ATG can decrease the incidence of DGF in comparison with Basiliximab in kidney transplant recipients with low immunological risk but high DGF risk.

**Methods:**

We conduct a phase IV, open label, randomized, multicentric and prospective study, to compare ATG in parallel with a control group treated by Basiliximab. The 1:1 randomized allocation of patients between groups is stratified on the clinical center, and on the hypothermic machine-perfusion device. We aimed to include a total of 384 patients to achieve a statistical power at 0.80. The study was initiated at the Nantes University hospital in July 2014, with data collection continuing until April 2018, and publication of the results proposed for 2019.

**Discussion:**

The main expected benefits of this study are *i*) the reduction of unjustified ATG over-prescriptions associated with serious adverse events, *ii*) the reduction of chance losses related to ATG under-prescription, *iii*) the decrease in the incidence of DGF which was described as a risk factor of graft failure and patient death, and *iv*) the reduction in hospitalization duration and number of post transplantation dialysis sessions, both being associated with reduced medical costs. In conclusion, the current study is innovative by proposing a more efficient and personalized induction therapy.

**Trial registration:**

The study was registered in the Clinical Trials Registry (#NCT02056938, February 5, 2014), and in the European Clinical Trials Database (EudraCT #2014-000332-42, January 30, 2014).

## Background

The current 10-year patient and graft survival of first kidney transplantation varies from 25 % to 40 % depending on inclusion criteria and countries. Therefore, much still has to be done to improve long-term outcomes for kidney transplant recipients. As part of this objective, delayed graft function (DGF) is well known among the main risk factors of poor long-term outcome, increased hospitalization duration and acute rejection [1, 2]. DGF is the major clinical manifestation of renal injuries that are linked to ischemia reperfusion injury. Until now, this has been unavoidable in kidney transplantation and occurs after cold and warm preservation of the kidney. Preventing DGF by focusing primarily on donor management, organ procurement, infusion and preservation supplies, and drug monitoring, is thus probably the most beneficial strategy for prolonged long-term graft and patient survival. Thus, the prediction of DGF is of primary importance in kidney transplantation for adapting as far as possible the medical management of high-risk transplantations.

However, although the risk factors for DGF have been well-described in the literature for a number of years, it is difficult to predict this complication in practice at an individual level. Several scoring systems have been proposed within the last few years [3, 4], but these are not used routinely for medical decision making because of the important number of included parameters or the absence of validation. Therefore, we recently proposed an alternative scoring system entitled the delayed graft function score (DGFS) [5]. The DGFS is composed of only five variables: the cold ischemia time, the donor age, the last donor serum creatinine level, the recipient body mass index, and the induction therapy used. The DGFS is associated with a good predictive capacity, with area under the receiver operator characteristic (ROC) curve at 0.73 (95 % CI from 0.68 to 0.77). By using the learning sample, we calculated a DGFS threshold value at 0.4 to obtain a positive predictive value of 40 %, an acceptable percentage to consider recipients with a high risk of DGF (the overall percentage of DGF was 25.1 %). By using the validation sample, this decision rule corresponds to sensitivity of 47.5 % (95 % CI from 40.3 to 55.0), a specificity of 83.5 % (95 % CI from 79.5 to 86.9), a positive predictive value of 57.7 % (95 % CI from 48.5 to 64.5), and a negative predictive value of 77.9 % (95 % CI from 73.7 to 81.6).

The use of anti-thymocyte globulins (ATG, Thymoglobuline®, Sanofi) as induction therapy has already been described as a possible treatment for reducing the prevalence of DGF [6–8]. ATG possesses pharmaceutical proprieties that could help to control or prevent the lesions caused by ischemia reperfusion injury. However, other studies have questioned this possible protective effect [9–12]. Therefore, despite this well-established depleting treatment as an induction therapy in patients with high immunological risk [13], there is no consensus on whether ATG is better than basiliximab (anti IL2-R, Simulect®, Novartis) to prevent DGF for patients with a low immunological risk. We hypothesized that the benefit related to ATG for reducing DGF prevalence may be higher than expected if only proposed to patients with high DGF risk.

The main objective of the PREDICT-DGF study outlined here is to determine whether a short course of ATG is more efficient than basiliximab in decreasing the incidence of DGF in kidney transplant recipients with low immunological risk, but high DGF risk assessed by the DGFS calculation. We also aim to evaluate the risk/benefit ratio related to induction therapy by ATG in patients with low immunological risk, i.e., the consequences of a possible reduction in DGF incidence, against adverse effects related to such depleting induction therapy.

## Methods/design

### Study design

**Fig. 1:**
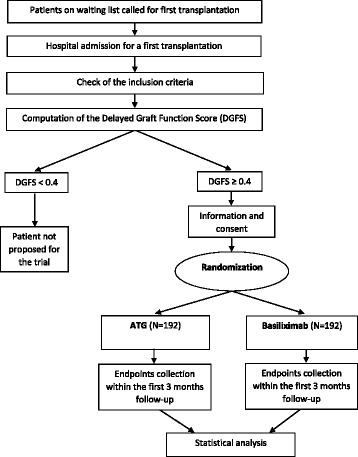
Preventing delayed graft function by driving immunosuppressive induction treatment (PREDICT-DGF) study: description of the study. This randomized trial aims to compare anti-thymocyte globulin (*ATG*)-treated patients in parallel with a control group treated by basiliximab. The 1:1 randomized allocation of patients between groups is stratified on the clinical center, and on the hypothermic machine-perfusion device. The participation of each patient is scheduled for 3 months

We are conducting a phase IV, open label, randomized, multicenter prospective study, to compare ATG in parallel with a control group treated by basiliximab. The 1:1 randomized allocation of patients between groups is stratified on the clinical center, and on the hypothermic machine-perfusion device. The participation of each patient is scheduled for 3 months. The study design is outlined in Fig. 1.

### Ethical approval and registration

All procedures will be explained to patients and the informed consent form will be reviewed and signed. The study was registered in the Clinical Trials Registry (#NCT02056938), and in the European Clinical Trials Database (EudraCT #2014-000332-42). It has received approval from the ethical research committee (Tours, France, Comité de Protection des Personnes – CPP, reference # 14/13-929) that is applicable for all French centers involved in this study. The French national agency for drugs safety also approved the study (Agence Nationale pour la Sécurité du Médicament, ANSM # 140175A-11), especially regarding the classification as a phase IV study. ATG and basiliximab have both been approved for marketing and the present protocol respects the corresponding indications.

### Study population

Recruitment is performed during pre-surgery hospitalization in one of the six participating centers (Lyon, Montpellier, Nancy, Nantes, Nice, and Paris Necker). Inclusion criteria are: 1) adults with end-stage renal disease; 2) patients under hemodialysis (because the occurrence of DGF, defined by the need for dialysis within the first seven days post transplantation, is not correctly evaluated for patients on peritoneal dialysis or for patients who will receive pre-emptive transplantation); 3) patients with kidneys transplanted from deceased donors with heartbeat; 4) patients with written informed consent; 5) patients affiliated with the French social security system; 6) patients considered at low immunologic risk (i.e., first kidney transplant recipients with no anti-HLA immunization prior to transplantation and sera negative for anti HLA immunization, regardless of the technique used for this evaluation); 7) patients considered at high-risk of DGF (i.e., DGFS higher than 0.4), and 8) patients without medical contraindication to ATG (i.e., leukopenia lower than 3,000/mm^3^, thrombopenia lower than 100,000/mm^3^, previous history of cancer (except basal cell skin cancers), current history of hepatitis C virus (HCV)/hepatitis C virus (HBV)/HIV infection, or Epstein-Barr virus (EBV)-matching donor positive and recipient negative). For ethical considerations, pregnant or lactating women and patients under guardianship are not included.

### Calculation of delayed graft function score

The DGFS calculation is facilitated by an application available on smartphones, tablets or computers at www.divat.fr/en/online-calculators. The results are exportable as a simple file in portable document format (PDF).

### Studied treatment (ATG)

Thymoglobuline® is used in this study. It is a rabbit anti-human thymocyte immunoglobulin. The first infusion begins prior to kidney reperfusion, to study the potential effects on ischemia reperfusion injury mechanisms [14–16]. In fever or respiratory syndrome related to possible cytokine release, paracetamol (1 g every 6 h) is prescribed, and Polaramine® (2 mg orally) prescribed in patients with associated cutaneous rash. The total duration of Thymoglobuline® administration is 4 days, including the first day of the surgery, with a daily dose of 1.5 mg/kg [17]. The monitoring of blood T cell depletion provides information for the adaptation of the Thymoglobuline® daily dose. The aim is to maintain peripheral blood lymphocytes below 200/mm^3^ and CD3 counts below 20/mm^3^. Thymoglobuline® dosing is halved if the white blood cell (WBC) counts falls below 2,000 − 3,000/mm^3^, or if the platelets fall in between 50,000 and 75,000/mm^3^. Thymoglobuline® is prematurely withdrawn if the WBC falls below 2,000/mm^3^ or the platelets fall below 50,000/mm^3^.

### Control treatment (basiliximab)

The control treatment is Simulect®, which is a recombinant murine/human chimeric monoclonal antibody directed against the interleukin-2 receptor α-chain (CD25 antigen). Two infusions of Simulect® are performed with a dose of 20 mg, the first immediately after surgery and the second 3 days afterwards.

### Other treatments

In parallel with Thymoglobuline® or Simulect®, the therapy includes one intravenous bolus of corticosteroids (methylprednisolone 500 mg). All patients receive the same immunosuppressive maintenance therapy with 1) Tacrolimus (Prograf®) regardless of the delayed administration post-surgery which respects normal practice at each center, and 2) mycophenolate mofetil (Cellcept®, with a daily dose of 2 g) and prednisolone (with a dose of 1 mg/kg orally tapered at 10 mg every 5 days to a dose of 5 mg until the surveillance biopsy at 3 months). Finally, concomitant prophylaxis by Trimethoprim/Sulfamethoxazole and Valgancyclovir will be used to prevent pneumocystis and cytomegalovirus (CMV) infection.

### Primary outcome measures

The primary endpoint will be the occurrence of a DGF defined as the need for dialysis within the first seven days post transplantation. This definition of DGF is the most commonly recognized and published. Nevertheless, this definition is insufficient since the decision to dialyze may not only depend on the insufficiency of the post-transplantation renal function but also on hyperhydration or hyperkaliemia for instance. The definition of the DGF will thus consider, 1) the number of days after transplantation to reach an estimated glomerular filtration rate (eGFR) above 10 mL/min, calculated using the modification of the diet in renal disease (MDRD) formula [18], 2) the evolution of the MDRD eGFR until hospital discharge, and 3) the number of dialyses performed after the transplantation without taking into account dialysis exclusively prescribed for hyperhydration or hyperkaliemia.

### Secondary outcome measures

The following endpoints will also be considered: 1) the occurrence of histologically proven acute rejection episodes within the 3 months following the transplantation and subclinical acute rejection episodes on the 3-month surveillance biopsies (BANFF 2011 classification); 2) the percentage of renal fibrosis on the 3-month surveillance biopsies using the computerized quantification described by Meas-Yedid et al. [19]; 3) the time between transplantation surgery and hospital discharge; 4) the evolution of the Tacrolimus trough level; 5) the hematologic monitoring within the first three months after surgery including WBC blood cell count, CD3 subpopulation analysis, and CD4, CD8, CD19, natural killer (NK) cell, and platelet counts, and 6) the occurrence of infections within the study follow-up, especially CMV and BK viruses assessed at three months by RT-PCR, as either primary or secondary infections.

### Follow-up time

Follow-up at 3 months is required for the previous endpoints for full completion of the study. Nevertheless, patients will be followed for some years after the end of the trial for incidence of cancer, post-transplant lymphoproliferative disorders, infections, graft and patient survival. This will be possible because these patients will be part of the prospective DIVAT cohort (www.divat.fr).

### Sample size

Based on the results of the randomized trial published by Noel et al. [13] and according to our previous observational study [5], we can expect 40 % DGF in patients treated with basiliximab versus 28 % in patients with ATG (odds ratio (OR) = 1.7). To respect a type I error at 0.05 and a power at 0.80 (one-tailed test), 192 patients will have to be included in each group. We therefore aim to include a total of 384 patients. We chose one-tailed testing because no paper has described a higher incidence of DGF for ATG-treated patients compared to other non-depleting induction therapies. The literature heterogeneity consists in the statistical significance of the ATG effect for preventing the DGF occurrence. Even if the two-tailed test takes into account the possibility that the reference treatment might be better [20], the one-tailed test seems the most adapted in our situation for both ethical and cost-efficiency considerations [21].

### Data acquisition

Data collection will be performed by the investigators or the clinical research assistants by using an electronic case report form accessible from the web and developed with Clinsight®. Data are anonymously extractable to keep the identities of patients confidential.

### Data analysis

Analysis will be performed by respecting the principle of intention-to-treat analysis. No intermediate analysis will be performed. Depending on the observed switches or dropouts, per-protocol analysis may be secondarily performed in order to evaluate the robustness of the findings. According to the sample size and the random assignment of treatments, both groups are expected to be comparable. In order to validate this comparability, a preliminary description of the two groups will be performed according to the mean, standard deviation and range for continuous variables, and according to percentage and effective for categorical variables. For binary endpoints, logistic regression with random intercept and/or slope will be performed to estimate the treatment effect, taking into account possible between-center differences. The same methodology will be used to assess the percentage of renal fibrosis and the time between surgery and hospital discharge. However, these will use linear models with random intercept and/or slopes. *P* <0.05 will be considered statistically significant for all tests. The statistical analyses will be performed using R software [22].

## Discussion

The study commenced at the Nantes University hospital in July 2014, and the inclusion of the other centers will be possible from September 2014, after the first phase of development in Nantes. It is reasonable to schedule a period of 42 months for patient recruitment, i.e., until January 2018, as guided by the number of transplantations usually performed among the six participating centers. Given the follow-up of 3 months, the end of the data collection is planned for April 2018 and the publication of results for 2019.

The objective of our study is to reduce the prevalence of DGF to improve the long-term outcomes of kidney transplant recipients. ATG is of potential interest to such a reduction, but the possible benefits are continually debated, probably due to the heterogeneity of its indication between countries, between centers, and even amongst physicians. Importantly, the main indication for this treatment is for patients with high immunological risk, i.e., patients having repeat transplantation and/or HLA immunization prior to transplantation). We hypothesized that the use of ATG in induction could be more beneficial in patients who present a low immunological risk but a high DGF risk. The use of the DGFS, to objectively identify patients with high DGF risk, is particularly important as it may allow heterogeneity practices to be aligned.

The consequences of the PREDICT-DGF study may be several-fold. One outcome may be to reduce excessive ATG prescription, which is associated with serious adverse events such as cytokine release syndrome, fever, serum sickness, infectious disease or post-transplant lymphoproliferative disorders. Another outcome may be to avoid ATG under-prescription and therefore to reduce the incidence of DGF. We also expect a shorter hospital stay, a reduction in the number of post transplantation dialyses, both being associated with a reduction of medical costs. Finally, this reduction may improve long-term graft and patient survival.

In conclusion, the current study is innovative as it will allow the effects of two types of induction therapy in kidney transplant recipients with low immunological risk to be evaluated, using for the first time a predictive tool to assess the risk of DGF. We believe this will result in a more efficient and personalized induction therapy.

## Trial status

The trial is ongoing.

## Abbreviations

CMV: cytomegalovirus
DGF: delayed graft function
DGFS: delayed graft function score
ATG: anti-thymocyte globulins
WBC: white blood cell
MDRD: modification of the diet in renal disease
eGFR: estimated glomerular filtration rate
PREDICT-DGF: preventing delayed graft function by driving immunosuppressive induction treatment
